# Variant allele frequency in circulating tumor DNA correlated with tumor disease burden and predicted outcomes in patients with advanced breast cancer

**DOI:** 10.1007/s10549-023-07210-9

**Published:** 2024-01-06

**Authors:** Jianxin Zhong, Hanfang Jiang, Xiaoran Liu, Hao Liao, Feng Xie, Bin Shao, Shidong Jia, Huiping Li

**Affiliations:** 1 Key Laboratory of Carcinogenesis and Translational Research (Ministry of Education/Beijing), Department of Breast Oncology, Peking University Cancer Hospital & Institute, Beijing, China; 2 Huidu Shanghai Medical Sciences, Shanghai, China

**Keywords:** Advanced breast cancer, Circulating tumor DNA, Liquid biopsy, Biomarkers, Prognosis

## Abstract

**Purpose:**

In patients with first-line advanced breast cancer (ABC), the correlation between ctDNA variant allele frequency (VAF) and tumor disease burden, and its prognostic value remains poorly investigated.

**Methods:**

This study included patients with ABC diagnosed at Peking University Cancer Hospital who performed ctDNA test before receiving first-line treatment. Baseline plasma samples were collected for assessing ctDNA alterations and VAF with next-generation sequencing. The sum of tumor target lesion diameters (SLD) was measured with imaging methods according to RECIST 1.1 criteria.

**Results:**

The final cohort included 184 patients. The median age of the cohort was 49.4 (IQR: 42.3–56.8) years. The median VAF was 15.6% (IQR: 5.4%-33.7%). VAF showed positive correlation with SLD in patients with relatively large tumor lesions (*r* = 0.314, *p* = 0.003), but not in patients with small tumor lesions (*p* = 0.226). VAF was associated with multiple metastasis sites (*p* = 0.001). Multivariate Cox regression analysis showed that high VAF was associated with shorter overall survival (OS) (HR: 3.519, 95% confidence interval (CI): 2.149–5.761), and first-line progression-free survival (PFS) (HR: 2.352, 95%CI: 1.462–3.782). Combined VAF and SLD improved prediction performance, both median OS and PFS of patients in VAF(H)/SLD(H) group were significantly longer than VAF(L)/SLD(L) group (mOS: 49.3 vs. 174.1 months; mPFS: 9.6 vs. 25.3 months).

**Conclusion:**

ctDNA VAF associated with tumor disease burden, and was a prognostic factor for patients with ABC. A combination of ctDNA test and radiographic imaging might enhance tumor burden evaluation, and improve prognosis stratification in patients with ABC.

**Supplementary Information:**

The online version contains supplementary material available at 10.1007/s10549-023-07210-9.

## Introduction

Breast cancer (BC) is the most prevalent tumor disease and the leading cause of death in women [1, 2]. Despite that the prognosis of early-stage breast cancer patients has been dramatically improved in recent decades, advanced breast cancer (ABC) is still intractable and presents poor clinical outcomes, which is characterized by metastasis disease, aggressive clinical behavior, and complex genomic landscape [3, 4]. There is an increasing emphasis on optimal tumor burden measurement and the importance of prognostication in advanced breast cancer clinical management. Although a variety of clinical features have been identified as markers of disease extension and predictors of prognosis, their discriminative ability remains limited [5]. Thus, there is a clinical need for new surrogate markers of tumor disease burden to be implemented for the clinical management of patients with advanced breast cancers.

ctDNA test has been widely used in precision oncology as a minimally invasive and rapid approach to picture genomic landscape in the setting of tumor disease, and applied in clinical practice for many purposes including monitoring treatment response and guiding treatment options [6–8]. Recently, an increasing number of studies have demonstrated that ctDNA VAF, which is the number of mutant molecules over total number of wild-type molecules at a specific location in the genome, could serve as a novel proxy for tumor burden and was associated with the prognosis of patients with cancer diseases [9–13]. On the other hand, traditional tumor markers such as serum CA15-3 and radiological parameters such as the Response Evaluation Criteria in Solid Tumors (RESIST) defined sum of the target lesion diameters were utilized in the clinical practice to measure the tumor disease burden and response to treatment [14–18]. Interestingly, whether there were significant correlations among these different kinds of tumor markers, and their predictive performance remains controversial. For instance, previous studies demonstrated that ctDNA VAF was positively correlated with CEA and tumor disease burden in metastatic colorectal cancer [19]. But Paolo Manca et al. revealed that, in metastatic colorectal cancer, ctDNA VAF was more efficient in OS prediction compared to CEA and RECIST-defined tumor lesion diameters, and the ctDNA VAF was significantly correlated with CEA but not with tumor lesion diameter [20]. In addition, Marin Strijker et al. reported that ctDNA VAF was significantly correlated with CA19.9 and tumor disease burden, and could effectively predict overall survival in metastatic pancreatic ductal adenocarcinoma [21]. However, in ABC setting, the prognostic value of ctDNA measured VAF, and the link between VAF and tumor disease burden has not been established.

In the present study, we aim to investigate the clinical value of VAF in ctDNA as a prognostic marker for patients with ABC, and the correlation between VAF and other tumor markers commonly available in the clinical practice, namely, CA15-3 and RECIST-defined sum of tumor target lesion diameters, to advance the application of ctDNA test in advanced breast cancer management.

## Materials and methods

### Patient cohort and clinical data collection

Patients diagnosed with metastatic relapse or de novo Stage IV metastatic breast cancer at Peking University Cancer Hospital between January 2018 and June 2022 who consented to perform ctDNA test were included in this study. The inclusion criteria were as follows: (1) female patients with ABC, (2) performed ctDNA test with baseline blood sample before first-line treatment, (3) has complete clinical pathological data, and (4) with measurable lesions present based on RECIST 1.1 criteria. The exclusion criteria were as follows: (1) male patients, (2) patients did not perform ctDNA test at baseline, (3) ctDNA samples failed to pass the quality control, (4) incomplete clinicopathological information available, and (5) only non-measurable tumor lesions present. All procedures involving human participants were approved by the Peking University Cancer Hospital ethical committee (No.2016KT75), and all patients provided written informed consent prior to blood collection for ctDNA test. All patients received the current standard therapies according to the NCCN clinical guideline [22]. The clinical information collected in this study included receptor status (estrogen receptor (ER), progesterone receptor (PR) that evaluated immunohistochemistry (IHC), and human epidermal growth factor receptor 2 (HER2)), histological type of primary tumor, age, primary tumor grade, Ki-67, primary TNM stage, progression-free survival (PFS), overall survival (OS), number of metastasis site, visceral metastasis status, and the sum of RECIST defined tumor lesion diameters. The last follow up was in July 2023. Genomic and clinical data of MSK-MET project were download from cBioPortal database (https://www.cbioportal.org/).

### Evaluating tumor disease burden according to RECIST criteria

Tumor size measurement was performed by computerized tomography (CT) or magnetic resonance imaging (MRI). Scans were evaluated by a radiologist according to RECIST 1.1 criteria [23]. The lesions with the longest diameters of > 10 mm were considered measurable target lesions, and lymph nodes were included if the short axis was > 15 mm according to the definitions for pathological lymph nodes reported in the RECIST 1.1 criteria. We evaluate the largest measurable lesions with a maximum of two lesions per organ, and a maximum of five lesions per patient. Tumor disease burden was then measured by calculating the total sum of measurable target lesion diameters (SLD).

### Sample collection and DNA extraction

Baseline plasma samples were collected from all 184 patients to analyze the genomic alterations of ABC. Metastatic tumor biopsies were obtained from 23 of the 184 patients to validate the concordance of alterations between plasma sample and tumor tissue. Blood samples were processed within 1 h after collection and stored at −20 °C until analysis. Frozen blood samples were thawed and centrifuged at 820 × g for 10 min. The supernatant was removed, centrifuged at 16,000 × g for 10 min, and the resulting supernatant was removed and stored at −80 °C. cfDNA was extracted from the plasma using QIAamp Circulating Nucleic Acid kit (Qiagen, Germantown, MD) and the quantity and quality of the purified cfDNA were checked using a Qubit dsDNA High Sensitivity kit and Bioanalyzer 2100 (Agilent, Santa Clara, CA. US). For samples with severe genomic contamination from peripheral blood cells, a bead-based size selection was performed to remove large genomic fragments. cfDNA was quantified using the LINE1 real-time PCR assay and stored at −20 °C.

### Sequencing library construction and sequencing

The harmonized 152-gene PredicineCARE™ NGS assay was performed at the College of American Pathologists (CAP) accredited laboratory at Huidu Shanghai Medical Sciences, Ltd. for detecting genomic alterations. The genes covered by this panel were listed in Supplementary Table 1. Purified cfDNA (from 1 to 2 mL plasma per sample) was subjected to adapter ligation, PCR amplification, and library construction. The quality and quantity of the amplified DNA libraries were checked using a Bioanalyzer 2100 to ensure that all samples had a main peak at ~ 300 base pairs (bp). Libraries were enriched with the PredicineCARE research panel using a hybrid capture method and deep sequenced by paired-end 2 × 150 bp sequencing on an Illumina paired-end 2 × 150 bp system on the Illumina NovaSeq 6000 sequencer with S4 flowcell [24, 25].

### Sequencing data analysis

The sequencing data were analyzed in-house using a custom NGS analysis pipeline. Briefly, paired-end reads originating from the same molecules were merged as single-strand fragments. Single-strand fragments from the same double-stranded molecules were further combined as double-stranded DNA. Both sequencing and PCR errors were deeply suppressed during this process. Detected variants were filtered based on variation background (compared with normal plasma samples and internal sample pools), repeat regions, and other quality metrics. The clinical significance of detected variants were annotated based on the Clinvar database. The LOD (limit of detection) is 0.25% for SNV and 2.23 for CNV. More details of the data analyzing processes were shown in Supplementary Methods.

### Variant calling

The process included adapter trimming, barcode checking, and correction. Cleaned, paired FASTQ files generated by the pipeline were further aligned to the human reference genome build hg19 using the Burrows-Wheeler Aligner (BWA) alignment tool. Consensus binary alignment map (BAM) files were derived by merging paired-end reads originated from the same molecules as single strand fragments, those from complementary double strand DNA molecules were further merged as double stranded. Single nucleotide variants (SNVs), small insertions and deletions (Indels), and copy number variations (CNVs) were identified across the targeted regions covered by the panel.

### Statistical analysis

Patients were stratified according to VAF, CA125, and SLD using median value as cut-off. According to previously studies, the highest VAF among all the mutations detected in one sample was selected to represent the VAF of the patient [19, 20]. Categorical data are presented as numbers and percentages, while the continuous data were described as medians and interquartile range (IQR). Cohen’s kappa was used to measure the concordance of variants between plasma and tumor tissue [26]. Fisher’s exact and Chi-square tests were used to compare the distribution of patients with defined clinicopathologic variables across subgroups divided by VAF, SLD, or CA15-3 levels. Kaplan–Meier curves and log-rank test were used to analyze patient outcomes including progression-free survival (PFS) and overall survival (OS). Spearman’s correlation analysis was used to measure the correlation among continuous variables. A univariate Cox regression model was performed to compute corresponding hazard ratios (HRs) and 95% confidence intervals (CI) for prognostic variables; variables with a *p* value < 0.1 were used to build multivariate models. Receiver operating characteristic (ROC), and corresponding area under curve (AUC) was applied to describe the predictive performance of variables. All tests were two-sided and a *P* value of < 0.05 was considered statistically significant. SPSS 25.0 and R 4.1 software were used for statistical analysis.

## Results

### Baseline characteristics of the ABC patients

**Fig. 1:**
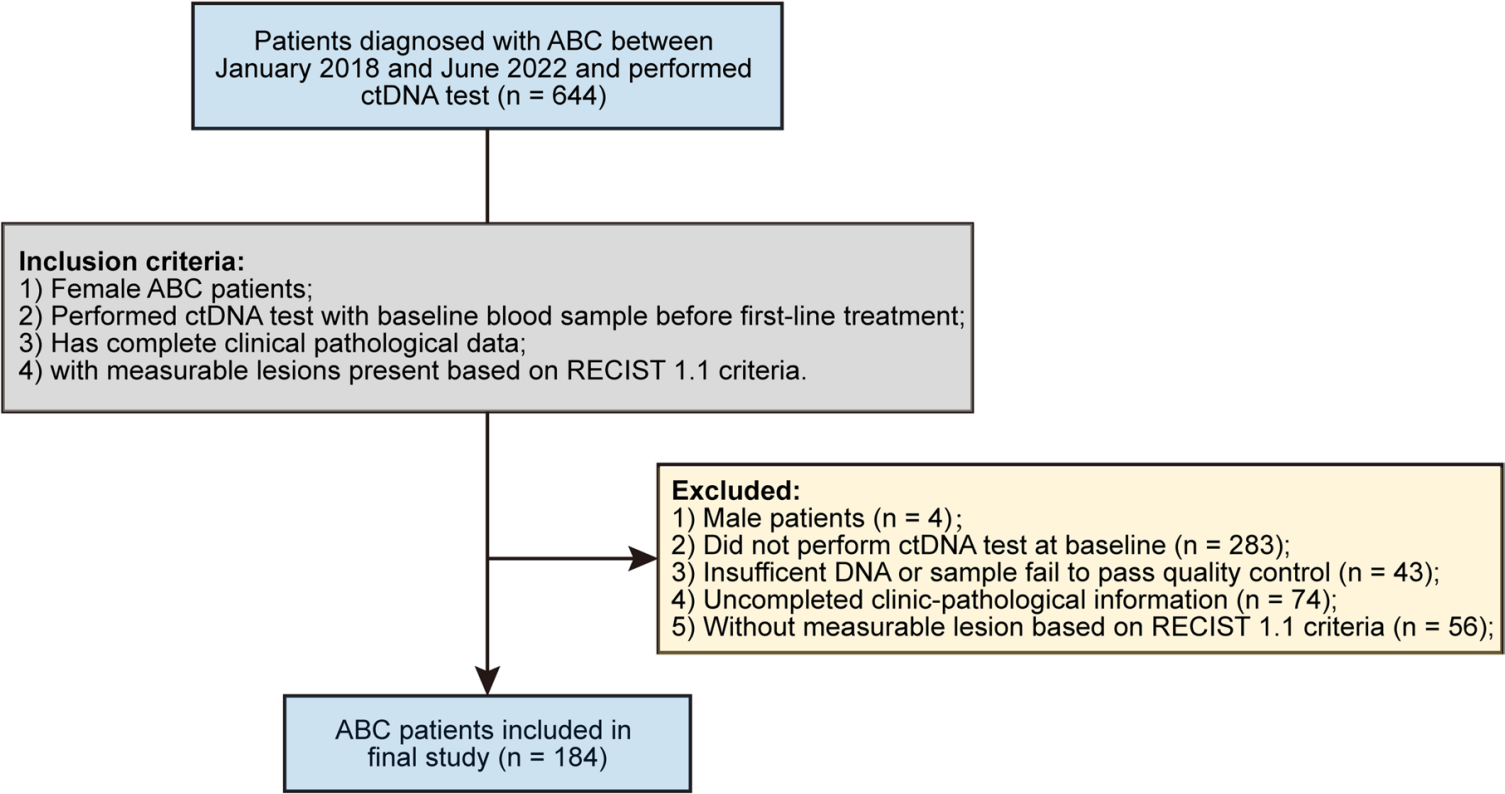
Consort diagram

A total of 184 women patients with ABC were included in this study (Fig. 1). The median age at diagnosis was 49.4 years (IQR: 42.3–56.8 years). Of all the 184 patients, 101 (54.9%) were HR+/HER2-, 30 (16.3%) were HR+/HER2+ , 24 (13.0%) were HR-/HER2+ , and 29 (15.8%) were triple-negative. With regard to metastases, 107 (58.2%) patients presented bone metastasis, 110 (59.8%) patients had lymph node metastasis, and only 5 (2.7%) had brain metastasis. Among all the ABC patients, most of them present more than one metastasis sites (132/184, 71.7%), and more than half of the patients had visceral metastasis (108/184, 58.7%). The median sum of tumor target lesion diameters (SLD) was 40.0 mm (IQR: 22.5–68.0 mm). The CA15-3 level in ABC patient was also elevated at baseline, with a median level of 59.4 U/ml (IQR: 36.0–127.5 U/ml).

### ctDNA alterations in advanced breast cancer

**Fig. 2:**
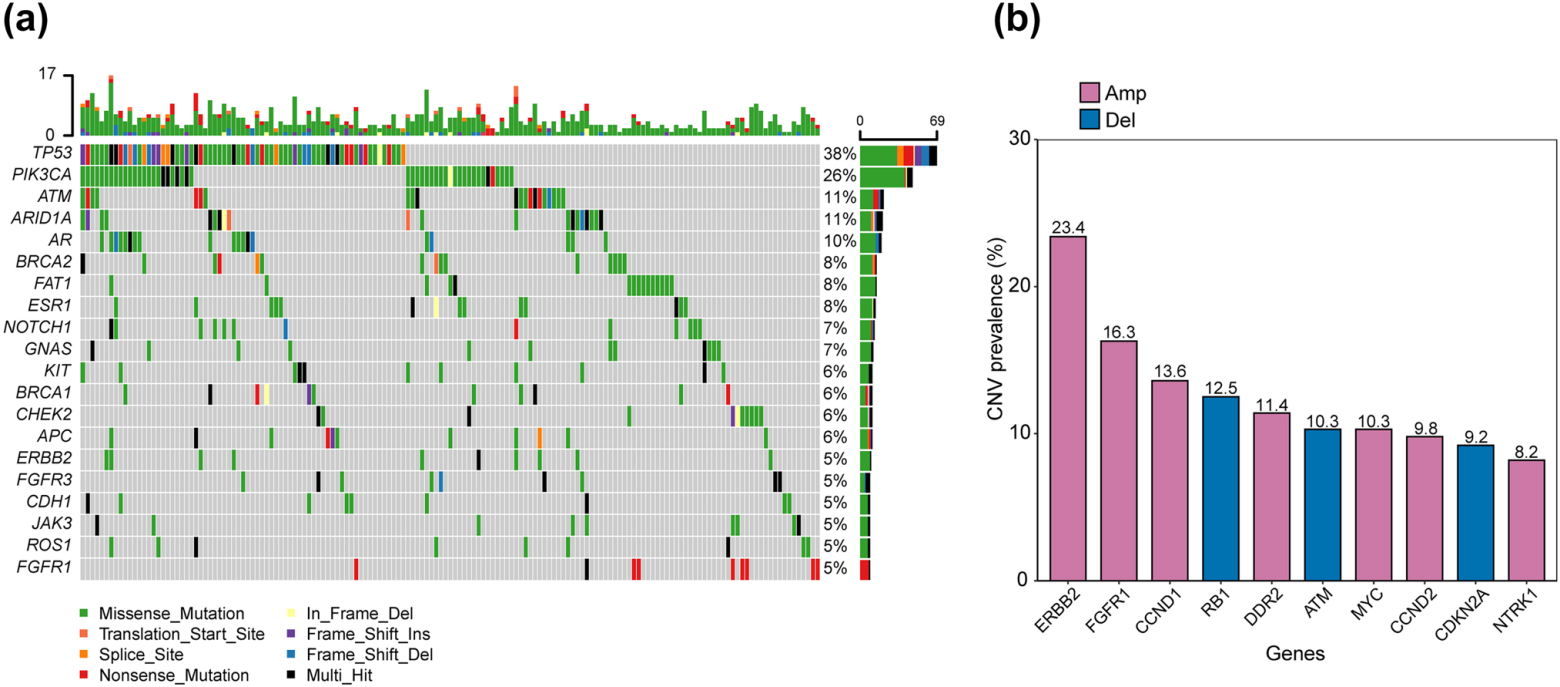
Genomic landscape of advanced breast cancer in circulating tumor DNA analysis. **(a)** Distribution and number of the top 20 SNVs by patient; **(b)** The prevalence of top 10 CNVs in ABC patients

Among all the patients, 171 (91.8%) patients occurred at least one SNV in ctDNA, who were available for VAF measurement. The landscape of genomic alterations for the entire cohort has been analyzed, and we found that the top 5 most frequently mutated genes among all patients were *TP53* (38%), *PIK3CA* (26%), *ATM* (11%), *ARID1A* (11%), *AR* (10%). The top 20 mutated genes were showed in Fig. 2a. The median VAF measured based on ctDNA samples of present cohort was 15.6% (IQR: 5.4%-33.7%) (Table 1). *TP53* was the gene with the highest VAF in approximately half of the cohort (30 out of 69 patients, 43.5%), followed by *PIK3CA* (17/48, 35.4%) (Supplementary Fig. 1). While CNV of at least one gene was detected in 109 (59.2%) patients. The most frequently detected CNVs of the top 10 frequently altered genes were shown by the barplot (Fig. 2b). The Kappa tests were performed to test evaluate the consistency between ctDNA and tissue samples (Supplementary Fig. 2a, d). TP53 SNVs were found in eleven tissue samples and seven plasma samples, with a match number of seven (kappa = 0.646; Supplementary Fig. 2b). Six tissue samples and five plasma samples present PIK3CA SNVs, and four pairs of samples had the same variants (kappa = 0.641; Supplementary Fig. 2c). Additionally, ERBB2 CNVs were detected in six tissue samples and six plasma samples, with a match number of five (kappa = 0.744; Supplementary Fig. 2e).

**Table 1:** Characteristics of the study cohort

Characteristics	Number of patients (N, %)
Age at diagnosis, years (Median, IQR)	49.4 (42.3–56.8)
Histological type at primary diagnosis	
Ductal	176 (95.6%)
Lobular	4 (2.2%)
Others	4 (2.2%)
Primary tumor stage^a^	
I	19 (10.3%)
II	71 (38.6%)
III	48 (26.1%)
IV	41 (22.3%)
Unknown	5 (2.7%)
De novo stage IV	
Yes	41(22.3%)
No	138 (75.0%)
Unknown	5 (2.7%)
Primary tumor grades	
I	10 (5.4%)
II	121 (65.8%)
III	45 (24.5%)
Unknown	8 (4.3%)
Ki-67	
< 20%	52 (28.3%)
≥ 20%	132 (71.7%)
Immunohistochemistry at primary diagnosis	
HR+/HER2-	101 (54.9%)
HR+/HER2+	30 (16.3%)
HR-/HER2+	24 (13.0%)
TNBC	29 (15.8%)
Metastatic sites	
Chest wall	52 (28.3%)
Bone	107 (58.2%)
Liver	63 (34.2%)
Lung	66 (35.9%)
Lymph nodes	110 (59.8%)
Brain	5 (2.7%)
Visceral metastasis^b^	
Yes	108 (58.7%)
No	76 (41.3%)
Number of metastasis sites	
1	52 (28.3%)
> 1	132 (71.7%)
Sum of tumor lesion diameters(mm)	40.0 (22.5–68.0)
CA15-3(U/ml)	59.4 (36.0–127.5)
Variant allele frequency (VAF in %)	15.6% (5.4%-33.7%)

Continuous variables were presented as median with IQR (interquartile range); Categorical variables were presented as counts with percentages

^a^Eighth edition American Joint Committee on Cancer TNM stage

^b^Visceral metastasis includes liver, lung, and brain metastasis

### Correlations between VAF and tumor disease burden

**Fig. 3:**
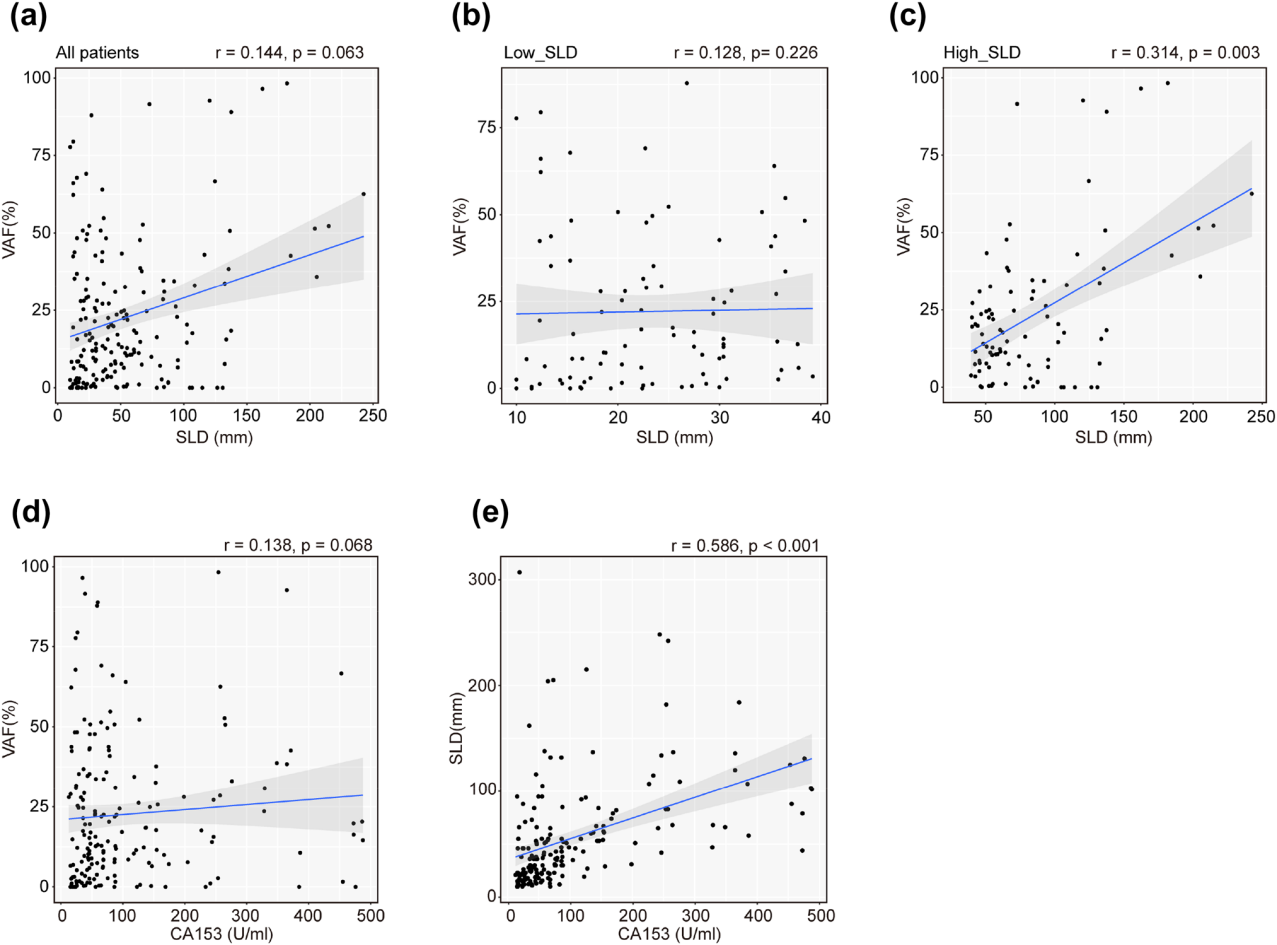
Correlation of VAF, SLD, and CA153. Scatter plots showing the correlations between VAF and RECIST-defined sum of the tumor target lesions (SLD) in the **(a)** overall cohort, **(b)** low_SLD group, and **(c)** high_SLD group. Correlation between **(d)** VAF and CA15-3, and **(e)** SLD and CA15-3. mm: millimeter, U/ml: units per milliliter

We next explored that whether VAF could serve as a surrogate of tumor disease burden by analyzing the correlation between VAF and traditional biomarkers including SLD and CA15-3. Among all the patients, VAF did not significantly correlated with SLD (Spearman’s *r* = 0.144, *p* = 0.063, Fig. 3a). Then, we divided ABC patients into high- and low-SLD groups by median value of SLD. In the low-SLD group, VAF was not significantly correlated with SLD (Spearman’s *r* = 0.128, *p* = 0.226, Fig. 3b), but presented a positive correlation in high-SLD group (Spearman’s *r* = 0.314, *p* = 0.003, Fig. 3c). Moreover, we found that VAF was not significantly correlated with CA15-3 (Spearman’s *r* = 0.138, *p* = 0.068, Fig. 3d) in ABC patients, while the CA15-3 showed a significantly positive correlation with SLD (Spearman’s *r* = 0.586, *p* < 0.001, Fig. 3e).

### Associations between tumor biomarkers and clinical features

We further explored the relationship between clinical characteristics and median VAF, CA15-3, and SLD in patients with ABC. Table 2 showed the difference in distribution of patients with high or low VAF, CEA, and tumor target lesion diameters according to the clinical characteristics. No significant imbalance was observed in the distribution of VAF, SLD, and CA15-3 across different BC subtypes. Notably, patients with multiple metastasis sites were more likely had higher VAF and CA15-3 (*p* = 0.001). Higher SLD was significantly associated with visceral metastasis (*p* = 0.005) and liver metastasis (*p* < 0.001). Moreover, higher CA15-3 was correlated with visceral metastasis (*p* = 0.027) and liver metastasis (*p* = 0.017).

**Table 2:** Distribution of VAF, CA15-3, and RECIST-defined sum of tumor lesion diameters according to baseline clinical features

Baseline features		VAF	*P*		Tumor lesion diameter		*P*	CA15-3		*P*
		Low	High		Low	High		Low	High	
Age (years)	< 49	41	46	0.555	41	46	0.652	42	45	0.876
	≥ 49	51	46		50	47		49	48	
Immunohistochemistry at primary diagnosis	HR+/HER2-	54	47	0.542	49	50	0.940	41	60	0.653
	HR+/HER2+	16	14		16	14		16	14	
	HER2+	10	14		11	13		10	14	
	TNBC	12	17		15	14		12	17	
Tumor grade	I	3	7	0.119	5	5	0.653	5	5	1.000
	II	65	56		62	59		60	61	
	III	18	27		19	26		22	23	
	Unknown	6	2		5	3		4	4	
Ki-67	< 20%	22	30	0.253	28	24	0.559	30	22	0.215
	≥ 20%	70	62		63	69		61	71	
De novo stage IV	No	71	67	0.692	67	66	0.274	70	68	0.785
	Yes	19	22		16	25		19	22	
	Unknown	2	3		3	2		2	3	
Visceral metastasis	Yes	53	54	0.454	43	64	**0.005**	45	62	**0.027**
	No	39	38		48	29		46	31	
Number of metastatic sites	1	37	15	**0.001**	26	26	0.867	36	16	**0.001**
	> 1	55	77		65	67		55	77	
Bone metastases	Yes	55	52	0.838	48	59	0.128	51	56	0.758
	No	37	40		43	34		40	37	
Liver metastases	Yes	29	34	0.534	19	44	** < 0.001**	23	40	**0.017**
	No	63	58		72	49		68	53	
Lung metastases	Yes	34	32	0.878	29	37	0.334	29	37	0.334
	No	58	60		62	56		26	56	
Lymph node metastases	Yes	52	58	0.452	47	63	0.452	51	59	0.383
	No	40	34		44	30		40	34	
Brain metastases	Yes	1	4	0.364	0	5	0.072	2	3	1.000
	No	91	88		91	88		89	90	
Chest wall metastases	Yes	71	62	0.188	19	32	0.059	25	26	1.000
	No	21	30		72	61		66	67	

Statistically significant *p* < 0.05 is highlighted in bold

### Prognostic impact of ctDNA VAF in ABC patients

**Fig. 4:**
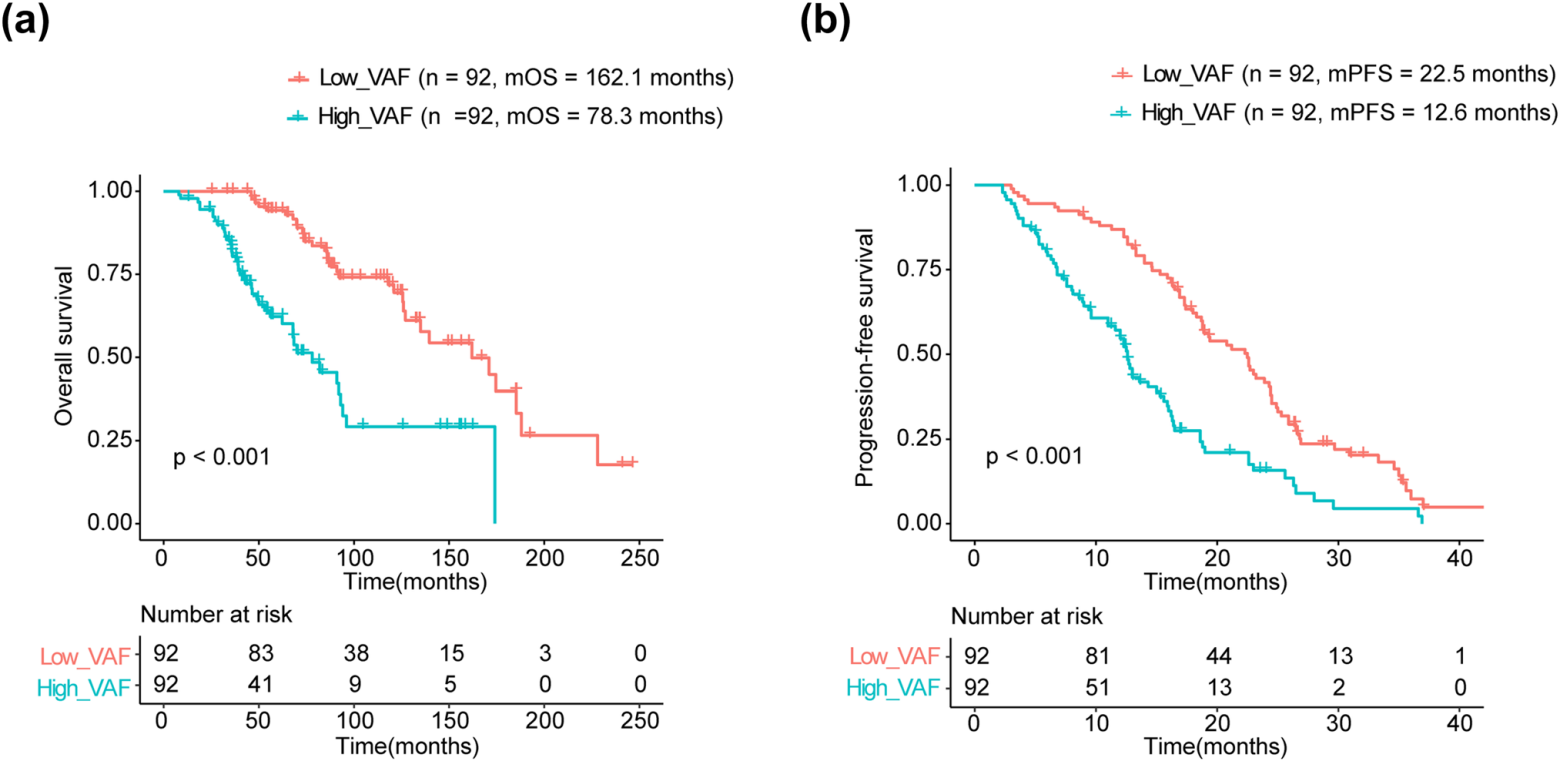
Survival analysis of **(a)** OS and **(b)** PFS between patients with high or low VAF levels

**Fig. 5:**
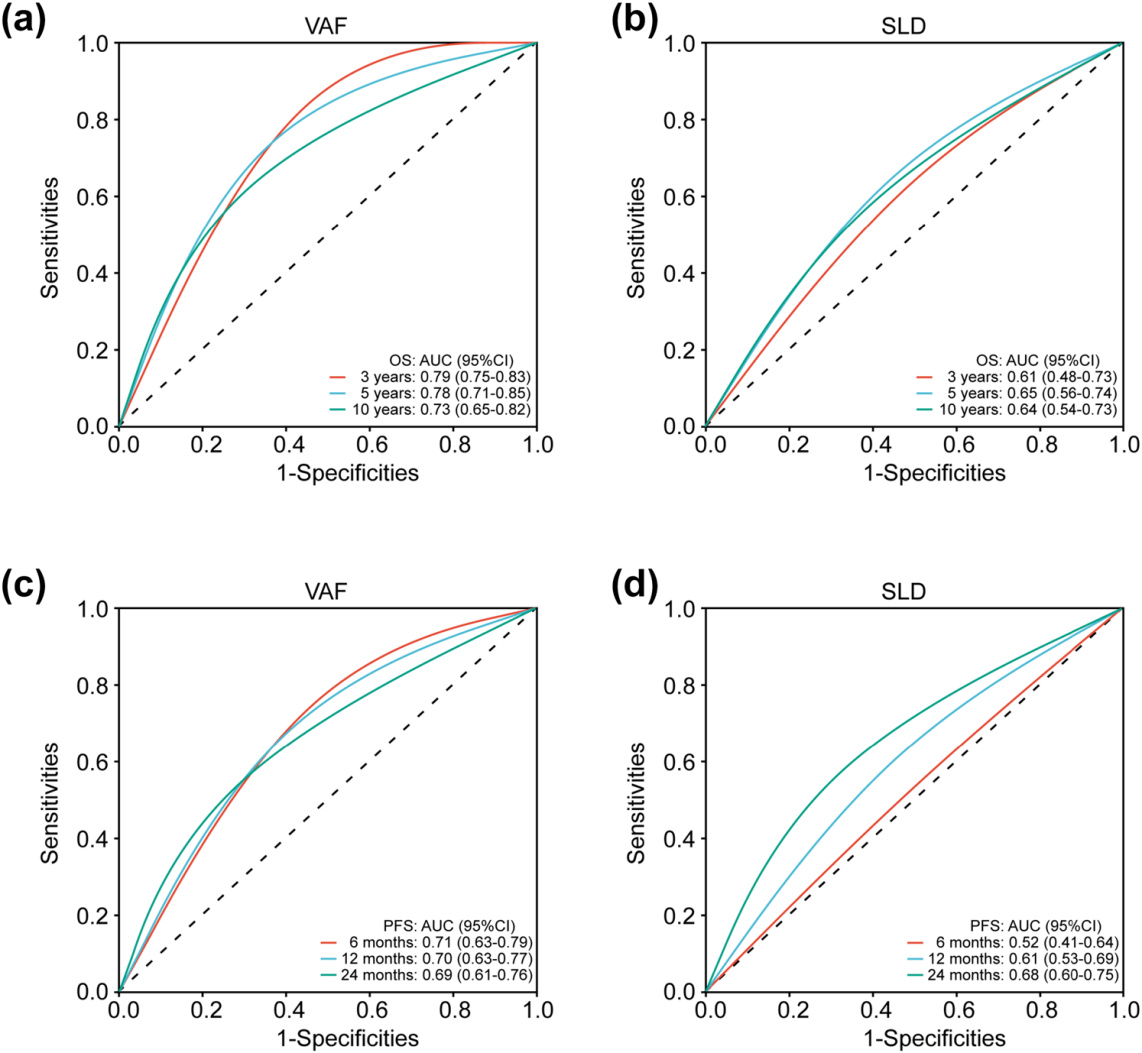
ROC curves of VAF and SLD for **(a, b)** OS and **(c**, **d)** PFS

We evaluated the prognostic impact of VAF in patients with ABC. Patients with higher VAF displayed a significantly poorer OS (mOS: 78.3 vs 162.1 months; HR: 3.519, 95%CI: 2.149–5.761; *p* < 0.001; Fig. 4a) and a significantly shorter PFS (mPFS: 12.6 vs 22.5 months; HR: 2.352, 95%CI: 1.462–3.782; *p* < 0.001; Fig. 4b). The results of univariate and multivariable Cox analysis for OS and PFS are shown in Table 3. Notably, VAF and SLD were significantly associated with both OS and PFS in the multivariable analyses, whereas CA15-3 was not (Table 3). In addition, the VAF was more efficient than SLD for predicting both OS (C-index: 0.687 vs. 0.598) and PFS (C-index: 0.626 vs. 0.584). The AUC values of ROC curves also indicated that predictive performance of VAF was better than SLD (5-years OS: 0.79 *vs.* 0.65; 12-months PFS: 0.70 *vs.* 0.65) which were higher than those of SLD and CA15-3 (Fig. 5a-d). The ROC curves also indicated VAF was more discriminative than CA15-3 (Supplementary Fig. 3a, b).

**Table 3:** Univariate and multivariate Cox regression analyses for OS and PFS

Variables		OS		PFS	
		HR (univariable)	HR (multivariable^b^)	HR (univariable)	HR (multivariable)
Age		1.003 (0.969–1.032), *p* = 0.994		1.042 (0.894–1.066), *p* = 0.476	
De novo stage IV	No	Ref.^a^		Ref	
	Yes	1.574 (0.927–2.672), *p* = 0.093	1.381(0.788–2.420), *p* = 0.258	1.174 (0.801–1.721), *p* = 0.411	
Primary tumor grade	I/II	Ref		Ref	
	IIII	1.157 (0.758–1.456), *p* = 0.244		1.235 (0.842–3.024), *p* = 0.372	
Ki-67	< 20%	Ref		Ref	
	≥ 20%	2.016 (0.825–4.174), *p* = 0.115		1.253 (0.746–2.104), *p* = 0.393	
Immunohistochemistry at primary diagnosis	HR + /HER2-	Ref		Ref	
	HR + /HER2 +	0.981 (0.624–1.544), *p* = 0.935		1.166 (0.923–2.658), *p* = 0.505	
	HER2 +	1.669 (0.941–3.005), *p* = 0.135		1.566 (0.923–2.658), *p* = 0.096	2.065 (0.814–3.396), *p* = 0.423
	TNBC	3.200 (2.041–5.018), *p* < 0.001	2.233 (1.134–4.396), *p* = 0.020	3.054 (1.953–4.775), *p* < 0.001	3.561 (2.213–5.732), *p* = 0.007
Visceral metastasis	No	Ref		Ref	
	Yes	1.280 (0.798–2.053), *p* = 0.305		1.505 (1.077–2.104), *p* = 0.017	1.307 (0.919–1.858), *p* = 0.137
Number of metastatic sites	Single	Ref		Ref	
	Multiple	0.554 (0.910–2.625), *p* = 0.099	1.220 (0.704–2.116), *p* = 0.478	1.578 (1.083–2.296), *p* = 0.017	1.696 (1.139–2.526), *p* = 0.009
Sum of tumor lesion diameters (SLD)^c^	Low	Ref		Ref	
	High	1.791(1.120–2.965), *p* = 0.015	2.040 (1.253–3.321), *p* = 0.004	1.991 (1.427–2.780), *p* < 0.001	1.782 (1.193–2.662), *p* = 0.005
CA15-3^c^	Low	Ref		Ref	
	High	1.064 (0.670–1.688), *p* = 0.794		1.538 (1.110–2.132), *p* = 0.009	1.249 (0.831–1.878), *p* = 0.285
VAF^c^	Low	Ref		Ref	
	High	3.518 (2.149–5.761), *p* < 0.001	3.323 (1.980–5.575), *p* < 0.001	2.352 (1.462–3.782), *p* < 0.001	1.979 (1.389–2.821), *p* < 0.001

^a^Ref.: Set as reference in Cox proportional hazards regression analysis

^b^Features with a *p* < 0.1 in the univariate analyses were used to build the multivariate models

^c^These features were considered as categorical variables and using median values as cut-off

**Fig. 6:**
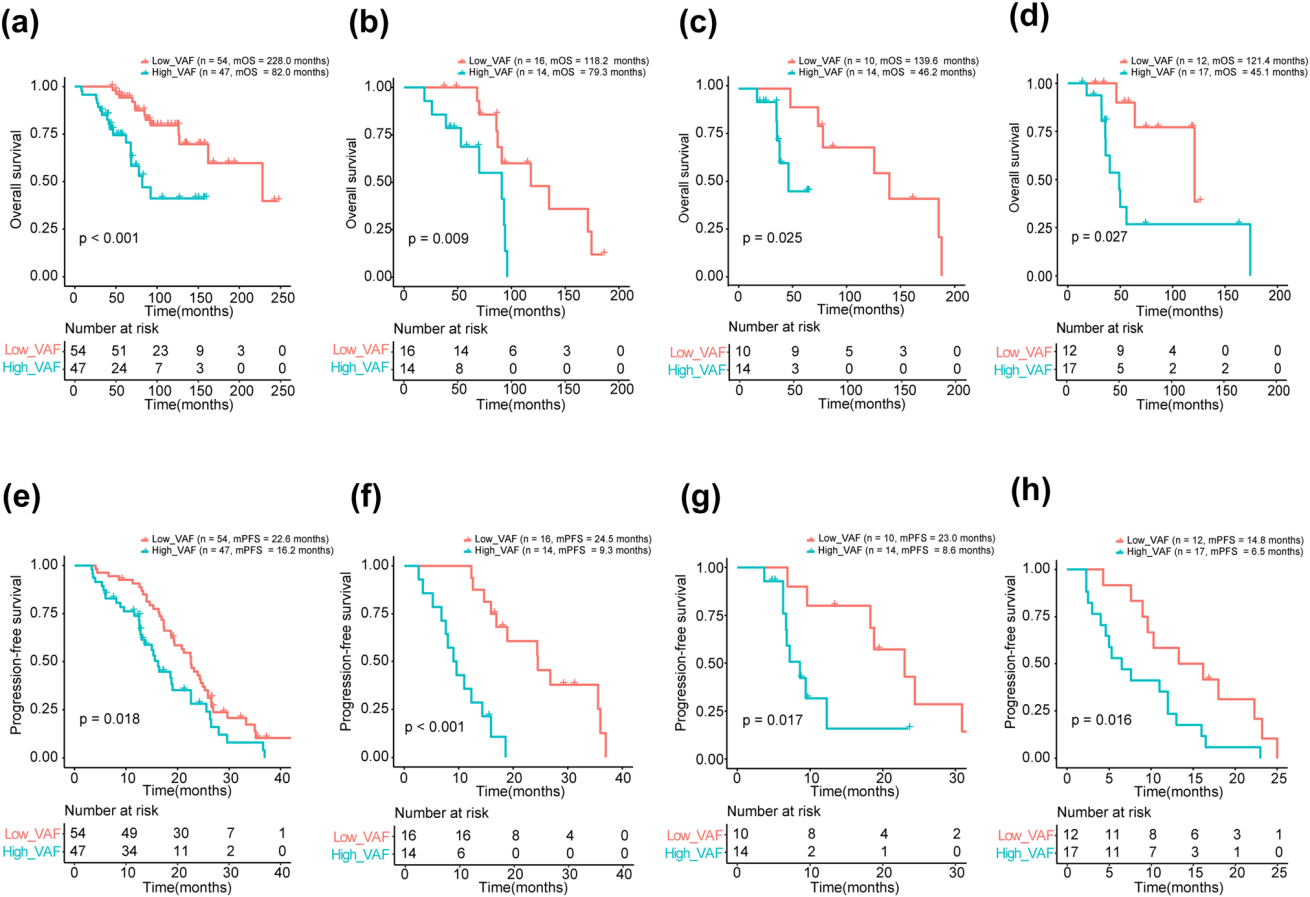
Survival analyses of OS and PFS among different BC subtypes. Kaplan–Meier curves of OS **(a-d)** and PFS **(e–h)** in patients with **(a, e)** HR + /HER2-, **(b, f)** HR + /HER2 + , **(c, g)** HR-/HER2 + , and **(d, h)** TNBC according to VAF levels;

Moreover, we assess the prognostic value of VAF in patients with four subtypes of BC. A significantly shorter OS was observed in the patients with a high VAF than those with a low VAF across four subtypes (HR+/HER2-: 82.0 vs. 228.0 months, Fig. 6a; HR+/HER2+ : 79.3 vs. 118.2 months, Fig. 6b; HR-/HER2+ : 46.2 vs. 139.6 months, Fig. 6c; TNBC: 45.1 vs. 121.4 months, Fig. 6d). The VAF remains predictive for PFS in four subgroups (Fig. 6e-h). These results indicated that ctDNA VAF was an independent prognostic factor, and showed stable predictive capacity in different BC subtypes. Additionally, we analyzed the prognostic value of VAF in patients with metastatic breast cancer from an external cohort (MSK-MET-MBC, *n* = 787, Supplementary Table 2). The patients in high-VAF group showed significant worse OS (HR: 1.572, 95%CI: 1.304–1.894; *p* < 0.001; Supplementary Fig. 4a), and obtained a satisfactory performance with the AUC value of 3- and 5-years OS was 0.70 and 0.72, respectively (Supplementary Fig. 4b).

### Combing ctDNA VAF with the RECIST-defined tumor lesion diameters improve prediction performance

**Fig. 7:**
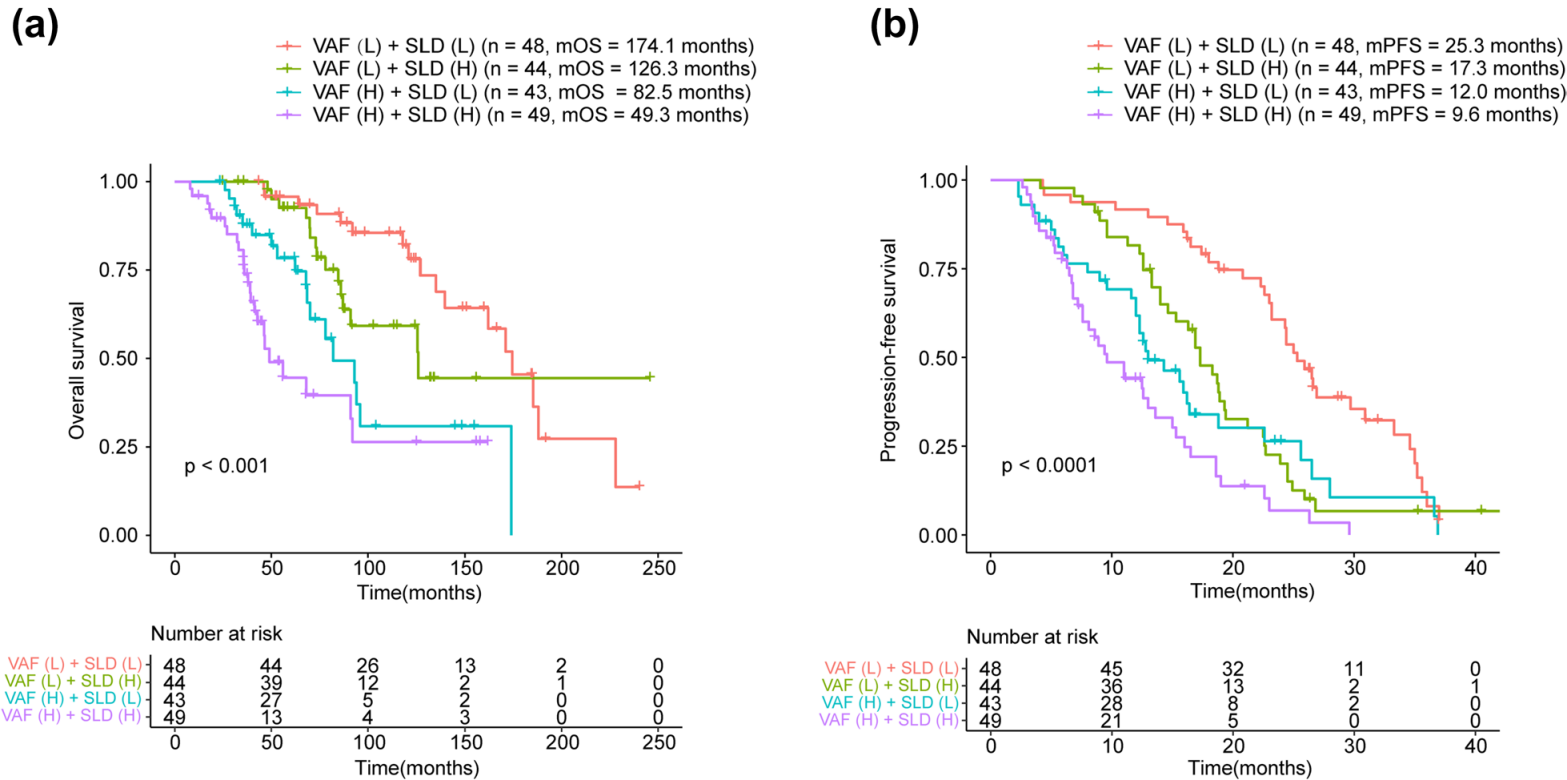
Prognostic value of combining VAF and SLD by Kaplan–Meier curves of **(a)** OS and **(b)** PFS

**Fig. 8:**
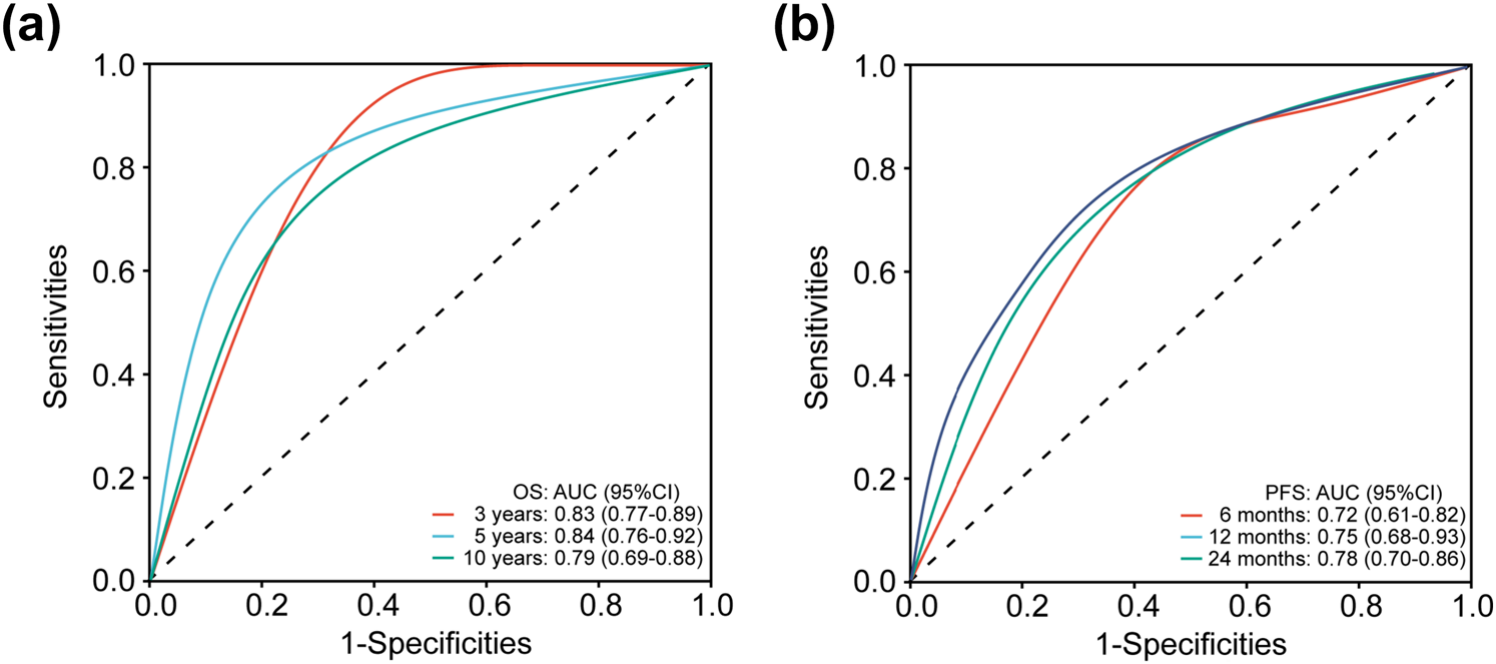
ROC curves of combining VAF and SLD for **(a)** OS and **(b)** PFS

The multivariate Cox regression model suggested that the baseline SLD and VAF were the most significant independent prognostic predictors for first-line PFS and OS of patients with ABC. We classified patients based on VAF combined with SLD. The results showed that VAF combined with SLD would effectively predict the clinical outcomes of patients. VAF(H)/SLD(H) patients (*n* = 49) had the worst prognosis, while VAF(L)/SLD(L) patients (*n* = 48) had the best prognosis (mOS: 49.3 vs. 174.1 months, HR: 5.710, 95%CI: 2.753–11.831, *p* < 0.0001; mPFS: 9.6 vs. 25.3 months, HR: 5.429, 95%CI: 3.186–9.245, *p* < 0.0001) (Fig. 7a, b). The ROC curves indicated that combining VAF and SLD of patients showed the high prediction accuracy (AUC: 0.84 for 5-years OS and 0.75 for 12-months PFS) (Fig. 8a, b).

## Discussion

Breast cancer present increasing incidence rate worldwide with high frequency of genomic alterations, ctDNA test was widely used for detecting therapeutic targets, predicting treatment response and clinical outcomes [27–29]. Recently, some studies reported the advantage of ctDNA test over classic imaging examinations such as CT and MRI in early detection of recurrence and progression of breast cancer, which challenging the efficiency of imaging methods in measuring tumor disease burden and risk stratification [30, 31]. To better understand the potential clinical impact of ctDNA test in advanced breast cancer, we evaluated variant allele frequency (VAF) in ctDNA and delve to its correlations with clinical characteristics, especially tumor disease burden. Moreover, we evaluated whether it could serve as a surrogate of disease burden and prognostic factor in comparison with RECIST-define sum of target lesion diameters and CA15-3.

Previous studies stratified the patients according to the presence or absence of mutations in ctDNA [32, 33] or used the total quantity of circulating-free DNA [21]. Recent studies also estimated the tumor burden by means of VAF in advanced solid tumors [20]. For instance, VAF could serve as both a prognostic biomarker and marker of tumor burden in metastasis colon cancer [9, 17, 34, 35]. But the role of VAF in ABC remains poorly investigated. In this study, VAF did not significantly correlated with SLD (*p* = 0.063) and CA15-3 (*p* = 0.137) in the overall cohort. Notably, in patients with lower SLD (defined as lower than median value of SLD), VAF was not correlated with SLD (*p* = 0.226), but in patients with relative higher SLD, VAF showed significant positive correlation with SLD (*r* = 0.314, *p* = 0.003). These results indicated that when tumor size is small, VAF could not efficiently reflect tumor burden, only in patients with larger tumor size, VAF might correlated with the tumor disease burden measured by imaging methods. Thus, the traditional imaging examination are still necessary to measure tumor size for early detection of primary tumor or recurrent tumor disease and monitoring the exact change of tumor lesion size for assessing treatment response. This work firstly provided real word evidence supporting that ctDNA VAF was unable to systematically evaluate tumor burden, and could not replace classical imaging approaches in clinical practice.

Clinical features such as number of metastasis sites or visceral metastasis are crucial for estimating tumor disease burden. ctDNA characteristics have also been demonstrated to be correlated with tumor metastasis in several studies. Zhang et al. reported that ctDNA-derived VAF was associated with lymph node metastasis in lung cancer [35], whereas Shibayama et al. reported that genomic variants in ctDNA from metastatic breast cancer patients did not correlate with visceral metastasis or the number of metastatic organs [36]. In contrast, Lam et al. found that ctDNA VAF was associated with visceral metastasis in advanced non-small-cell lung cancer patients [37]. In the present study, we found one significant correlation between VAF and the number of metastasis (Table 2). No associations were detected between visceral metastasis and VAF, indicating a limitation of VAF in depicting characteristics of patients with ABC. These findings supported that a comprehensive analysis of tumor disease should be multiple dimensions.

It has been proved that ctDNA VAF could efficiently predict the prognostic of patients with cancer diseases including lung and bladder cancers [9, 38, 39]. This study filled in a gap of prognostic role of VAF in advanced breast cancer. In this cohort, patients with higher VAF had significantly shorter PFS and OS when comparing to the low VAF group. Previous studies indicated that ctDNA VAF was more efficient than CEA and tumor target lesion in metastatic colorectal cancer, and more efficient than imaging methods measured tumor size in predicting lymph node metastasis in lung cancer [20, 35]. Similarly, in this study, ctDNA VAF also showed the optimal efficiency in predicting clinical outcomes of patients with ABC comparing to SLD and CA15-3 across four subtypes of BC. The significant association between VAF and prognosis of patients with ABC highlighting that VAF casting tumor disease burden based on individual genomic features, and provide disease information in a dimension different from imaging methods and traditional plasma biomarker. Previous study suggested that a combination with AFP could improve the sensitivity and specificity of ctDNA for predicting prognosis of patients with liver cancer [40]. Interestingly, we found that a combination of VAF and SLD reinforce the capacity of predicting prognosis of patients with ABC.

In conclusion, we found that ctDNA VAF at baseline could not precisely reflect tumor size alone, especially when tumor lesion is small, but correlated with multiple metastasis sites, shorter PFS and OS in patients with ABC. Moreover, a combination of the ctDNA test and imaging approaches, both of which could be rapidly assessed, might be optimal for systematically assessing tumor burden and predicting clinical outcomes, which presents translational relevance for potential clinical applications.

### Supplementary Information

Below is the link to the electronic supplementary material.

Supplementary file1 (DOC 155533 KB)

Supplementary file2 (DOC 30 KB)

## Data Availability

The data that support the findings of this study are available from the corresponding author upon reasonable request.

## Abbreviations

BC: Breast cancer
IHC: Immunohistochemistry
ER: Estrogen receptor
PR: Progesterone receptor
HER2: Human epidermal growth factor receptor 2
TNBC: Triple-negative breast cancer
FISH: Fluorescence in situ hybridization
DCIS: Ductal carcinoma in situ
IDC: Invasive ductal carcinoma
ctDNA: Circulating tumor DNA
SNV: Single nucleotide variants
Indels: Small insertions and deletions
CNV: Copy number variations
VAF: Variant allele frequency
SLD: Sum of target tumor lesion diameters
CA15-3: Cancer antigen 15–3
NCCN: National comprehensive cancer network
ROC: Receiver operating characteristic
AUC: Area under the ROC curve
PFS: Progression-free survival
OS: Overall survival
ROC: Receiver operating characteristic
AUC: Area under the curve
