# Supplementary material for: Variant allele frequency in circulating tumor DNA correlated with tumor disease burden and predicted outcomes in patients with advanced breast cancer
Source: Breast Cancer Res Treat. 2024 Jan 6;204(3):617–29. doi: 10.1007/s10549-023-07210-9 (PMC10959836; doi:10.1007/s10549-023-07210-9)
Supplement: Supplementary file 2 — Supplementary file2 (DOC 30 KB) [file 10549_2023_7210_MOESM2_ESM.doc]

**Supplementary Method**

**Sample collection and DNA extraction**

Each single draw of 10 mL of whole blood was collected into a Streck tube before undergoing a twostep centrifugation to separate plasma and buffy coat compartments. Aliquoted samples were stored at -80°C for batch processing. DNA extraction, library preparation and sequencing were performed in a CAP-accredited laboratory (Huidu Shanghai). Circulating cell-free DNA (cfDNA) was extracted from plasma samples using the QIAamp circulating nucleic acid kit (Qiagen, Hilden, Germany). Genomic DNA (gDNA) was extracted from matched peripheral blood mononuclear cells (PBMCs) using the QIAamp DNA Blood Mini Kit (Qiagen), then enzymatically fragmented and purified.

DNA is extracted by HiPure FFPE DNA kit from FFPE samples. Quantity and quality of the

extracted DNA are checked using Qubit fluorimeter and Tapestation 4200. Up to 500ng genomic DNAs are fragmented by bioruptor. Quantity and quality of the fragmented DNA are checked using Qubit fluorimeter and Bioanalyzer 2100.

**library preparation and capture**

Library preparation included DNA end-repair, dA-tailing and adapter ligation followed by polymerase chain reaction (PCR) amplification (eight cycles). The amplified DNA libraries then underwent quality control assessment on the Bioanalyzer 2100 and samples with adequate yield (>700 ng) were selected for hybrid capture.

Hybrid capture was conducted using biotin labelled DNA probes. In brief, the library was hybridized overnight with the PredicineCARE^TM^ panel (Predicine, Inc., CA, USA) and bead-captured beads. The unbound fragments were washed away, and the enriched fragments were amplified via PCR amplifications. The purified product was checked on a Bioanalyzer 2100 and then loaded into an Illumina NovaSeq 6000 (San Diego, CA, USA) for NGS sequencing with paired-end 2x150bp sequencing kits.

**Analyses of NGS data generated from cfDNA**

Consensus binary alignment map (BAM) files were derived by merging paired-end reads that originated from the same molecules (based on mapping location and unique molecular identifiers) as single strand fragments. Single-strand fragments from the same double-strand DNA molecules were merged to be double stranded for suppressing sequencing and PCR errors during this process. NGS quality-checking was performed by examining the percentage of targeted regions with >1500x unique consensus coverage. Samples with <80% regions having >1500x unique coverage were deemed to be QC failed and excluded. Candidate variants, consisting of point mutations, small insertions and deletions, were identified using the in-house developed pipeline across the targeted regions covered in the PredicineCare panel by comparing with local variant background [Newman 2016] (defined based on plasma samples from health donors and historical data).

**Analyses of NGS data from cfDNA**

NGS data from cfDNA were analyzed using the Predicine DeepSea NGS analysis pipeline, which starts from the raw sequencing data (BCL files) and outputs the final mutation calls. Briefly, the pipeline first performed adapter trimming, barcode checking, and correction. Cleaned paired FASTQ files were aligned to human reference genome build hg19 using the BWA alignment tool. Consensus BAM files were then derived by merging paired-end reads originated from the same molecules (based on mapping location and unique molecular identifiers) as single strand fragments. Single strand fragments from the same double strand DNA molecules were further merged as double stranded. By using the error suppression method described in [Newman, 2016], both sequencing and PCR errors were mostly corrected during this process.

**Identification of somatic mutations**

A variant identified in cfDNA was considered to be a candidate somatic mutation based if all of the following pre-defined criteria were present. These criteria were 1) the presence of at least 5 distinct paired reads in the mutation in the plasma; 2) the number of distinct paired reads containing a particular mutation in the plasma is at least 0.1% of the total distinct read pairs (if the nucleotide change and amino acid change are identical to an alteration observed in ≥20 cancer cases reported in the COSMIC database or previously reported as a cancer hotspot [http://www.cancerhotspots.org]) or the number of distinct paired reads containing a particular mutation in the plasma was at least 0.3% of the total distinct read pairs (if the nucleotide change and amino acid change are not a frequent alteration in COSMIC database or reported as a cancer hotspot previously); 3) the variant is not present in public databases of common germ line variants, including 1000 genomes, ExAC, gnomAD, and KAVIAR, with population allele frequency >0.5%; 4) the variant is not present in matched PBMC samples with allele frequency >5%(unpublished data, manuscript in preparation).

Candidate variants were called by comparing with local variant background (defined based on plasma samples from healthy donors and historical data). Variants were further filtered by log-odds (LOD) threshold [Cibulskis 2013], base and mapping quality thresholds, repeat regions and other quality metrics. Candidate somatic mutations were further filtered on the basis of gene annotation to identify those occurring in protein-coding regions. Intronic and silent changes were excluded, while mutations resulting in missense mutations, nonsense mutations, frameshifts, or splice site alterations were retained. Mutations annotated as benign or likely benign were also filtered out based on the ClinVar database [Landrum, 2016], or as common germline variants in databases including 1000 genomes [Auton, 205; Sudmant, 2015], ExAC [Lek, 2016], gnomAD (http://gnomad.broadinstitute.org) and KAVIAR [Glusman, 20111] with population allele frequency >0.5%. Finally, hematopoietic expansion-related variants that have been previously described, including those in DNMT3A, ASXL1, TET2, and specific alterations within ATM (residue 3008), GNAS (residue 201, 202), or JAK2 (residue 617) were marked as CHIP-related mutations.

**Reference**

1) Newman AM, Lovejoy AF, Klass DM et al. Integrated digital error suppression for improved

detection of circulating tumor DNA. Nat Biotechnol 2016; 34:547-555.

2) Cibulskis K, Lawrence MS, Carter SL et al. Sensitive detection of somatic point mutations in impure

and heterogeneous cancer samples. Nat Biotechnol 2013; 31:213-219.

3) Landrum MJ, Lee JM, Benson M et al. ClinVar: public archive of interpretations of clinically

relevant variants. Nucleic Acids Res 2016; 44:D862-868.

4) Auton A, Abecasis GR, Altshuler DM et al. A global reference for human genetic variation. Nature

2015; 526:68-74.

5) Sudmant PH, Rausch T, Gardner EJ et al. An integrated map of structural variation in 2,504 human

genomes. Nature 2015; 526:75-81.

6) Lek M, Karczewski KJ, Minikel EV et al. Analysis of protein-coding genetic variation in 60,706

humans. Nature 2016; 536:285-291.

7) Glusman G, Caballero J, Mauldin DE, Hood L, Roach JC. Kaviar: an accessible system for testing

SNV novelty. Bioinformatics 2011; 27:3216-3217.
