# Supplementary material for: Variant allele frequency in circulating tumor DNA correlated with tumor disease burden and predicted outcomes in patients with advanced breast cancer
Source: Breast Cancer Res Treat. 2024 Jan 6;204(3):617–29. doi: 10.1007/s10549-023-07210-9 (PMC10959836; doi:10.1007/s10549-023-07210-9)
Supplement: Supplementary file 1 — Supplementary file1 (DOC 155533 KB) [file 10549_2023_7210_MOESM1_ESM.doc]

**Supplementary Figure 1** The Barplot depicted the proportion of patients presented the highest VAF in TP53 or PIK3CA

**Supplementary Figure 2** Concordance analysis of variants between plasma and tissue samples

Concordance of **(a)** SNVs and **(b)** CNVs between tissue samples and plasma ctDNA. In the matrices, the top bar charts depict the number of mutations. Each column represent a patient. The text on the left represents gene names. Coloured triangles in the squares indicate mutations. If a square was filled with two coloured triangles, the same mutation was detected in the corresponding patient’s plasma and tissue. Overlapping **(b)** TP53 SNVs, **(c)** PIK3CA SNVs, and **(e)** ERBB2 CNVs between tissue and plasma ctDNA. SNVs, single nucleotide variations; CNVs, copy number variations; ctDNA, circulating tumour DNA

**Supplementary Figure 3** ROC curves of CA15-3 for **(a)** OS and **(b)** PFS

**Supplementary Figure 4 (a)** KM curves showed overall survival of patients with high- or low-VAF, and **(b)** ROC curves of VAF for OS in MSK-MET-MBC cohort.

Supplementary Table 1 Gene list of 152 gene panel used for cfDNA test in this study

| **Gene list of PredicineCARE panel** | | | | | | | | | |
| --- | --- | --- | --- | --- | --- | --- | --- | --- | --- |
| **Entire coding sequence and CNVs** | | | | | | | | | |
| ABRAXAS1 | AKT1 | AKT2 | AKT3 | ALK | APC | AR | ARAF | ARID1A | ATM |
| ATRX | BAP1 | BARD1 | BCL2 | BRAF | BRCA1 | BRCA2 | BRIP1 | BTK | CCND1 |
| CCND2 | CCND3 | CCNE1 | CCNE2 | CD274 (PD-L1) | CDH1 | CDK12 | CDK2 | CDK4 | CDK6 |
| CDKN2A | CHEK1 | CHEK2 | CTNNB1 | CXCR4 | CYP2C19 | CYP2D6 | CYP3A4 | DAXX | DDR2 |
| DPYD | E2F1 | EGFR | EPCAM | ERBB2 (HER2) | ERBB3 | ERCC1 | ESR1 | EZH2 | FANCA |
| FANCC | FANCF | FANCG | FANCL | FAT1 | FBXW7 | FEN1 | FGFR1 | FGFR2 | FGFR3 |
| FGFR4 | FLT3 | FOXA1 | FOXL2 | FZR1 | GEN1 | GNA11 | GNAQ | GNAS | GSTP1 |
| HNF1A | HOXB13 | HRAS | IDH1 | IDH2 | JAK2 | JAK3 | KDM6A | KIT | KMT2C |
| KMT2D (MLL2) | KRAS | MAP2K1 (MEK1) | MAP2K2 (MEK2) | MAPK1 | MAPK3 | MDM2 | MET | MLH1 | MPL |
| MRE11 | MSH2 | MSH6 | MTHFR | MTOR | MYC | MYCN | MYD88 | NBN | NF1 |
| NFE2L2 | NOTCH1 | NPM1 | NRAS | NTRK1 | NTRK2 | NTRK3 | PALB2 | PDCD1LG2 (PD-L2) | PDGFRA |
| PIK3CA | PIK3CB | PIK3R1 | PLCG2 | PMS2 | POLD1 | POLE | PPP2R1A | PRKACA | PRKD1 |
| PTEN | PTPN11 | RAD50 | RAD51 | RAD51B | RAD51C | RAD51D | RAD52 | RAF1 | RB1 |
| RET | RHEB | RHOA | RIT1 | RNF43 | ROS1 | SDHB | SMAD4 | SMO | SPOP |
| STAG2 | STK11 | TERT promoter | TMPRSS2 | TP53 | TSC1 | TSC2 | UGT1A1 | VHL | XPC |
| XRCC1 |  |  |  |  |  |  |  |  |  |
| **Fusions** | | | | | | | | | |
| ALK | BRAF | CD274 | CD74 | EGFR | FGFR1 | FGFR2 | FGFR3 | NTRK1 | NTRK2 |
| PDGFRA | PRKACA | RET | ROS1 | TMPRSS2 |  |  |  |  |  |

**Supplementary Table 2** Characteristics of the MSK-MET-MBC cohort

| **Characteristics** | **Number of patients (N, %)** |
| --- | --- |
| **Age at metastasis, years** (Median, IQR) | 55.8 (46.9-63.5) |
| **Histological type at primary diagnosis**  Ductal  Lobular | 644 (81.8%)  143 (18.1%) |
| **Immunohistochemistry at primary diagnosis**  HR+/HER2-  HR+/HER2+  HR-/HER2+  TNBC | 562 (71.4%)  79 (10.0%)  35 (4.4%)  111 (14.1%) |
| **Number of metastasis sites**  ≤ 1  > 1 | 100 (12.7%)  687 (87.3%) |
| **Metastatic sites**  Bone  Liver  Lung  Lymph nodes  Brain/central nervous system  Breast | 106 (13.5%)  222 (28.2%)  62 (7.8%)  114 (14.4%)  35 (24.4%)  8 (1.0%) |
| **Variant allele frequency (VAF in %)** | 20.7% (6.1%-41.7%) |
